# Failure-specific prognostic factors after continuous hyperfractionated accelerated radiotherapy (CHART) or conventional radiotherapy in locally advanced non-small-cell lung cancer: A competing risks analysis

**DOI:** 10.1054/bjoc.2001.2049

**Published:** 2001-10

**Authors:** Ö U Ataman, S M Bentzen, M I Saunders, S Dische

**Affiliations:** Gray Cancer Institute, Mount Vernon Hospital Northwood, Middlesex, UK; Marie Curie Research Wing, Mount Vernon Hospital Northwood, Middlesex, UK; Radiation Oncology Department, Dokuz Eylül University Medical School, Izmir, Turkey

**Keywords:** NSCLC, CHART, competing risks, failure-specific prognostic factors

## Abstract

The aim of this study was to identify possible failure-specific prognostic factors in non-small-cell lung cancer. Clinical outcome was analysed in 549 patients participating in the randomized controlled trial of CHART vs conventional radiotherapy. Local failure and distant failure with or without concurrent local relapse were subjected to a competing risk analysis using an accelerated failure-time model with a log-logistic hazard function. Randomization to CHART (2 *P* = 0.005), increasing age (2 *P* = 0.036) and female sex (2 *P* = 0.09) was all associated with a prolonged interval to failure. Advanced clinical stage was associated with a decreased interval to failure (2 *P* = 0.004) and a significantly increased risk (2 *P* = 0.009) of failing in distant rather than in local position. From this model, prognostic indices for local and distant failure were estimated for each individual patient. Competing risk analysis allows identification of patients with different failure patterns, and may provide a means of stratifying patients for intensified local or systemic therapy. © 2001 Cancer Research Campaign  http://www.bjcancer.com


*British Journal of Cancer* (2001) **85**(8), 1113–1118
© 2001 Cancer Research Campaign
doi: 10.1054/ bjoc.2001.2049, available online at http://www.idealibrary.com on IDEAL

### Failure-specific prognostic factors after continuous hyperfractionated accelerated radiotherapy (CHART) or conventional radiotherapy in locally advanced non-small-cell lung cancer: A competing risks analysis

**ÖU Ataman[1,3], SM Bentzen[1], MI Saunders[2] and S Dische[2]**

[1]Gray Cancer Institute, Mount Vernon Hospital Northwood, Middlesex, UK; [2]Marie Curie Research Wing, Mount Vernon Hospital Northwood, Middlesex, UK; [3]Dokuz Eylül University Medical School Radiation Oncology Department, Izmir, Turkey

**Summary** The aim of this study was to identify possible failure-specific prognostic factors in non-small-cell lung cancer. Clinical outcome was analysed in 549 patients participating in the randomized controlled trial of CHART vs conventional radiotherapy. Local failure and distant failure with or without concurrent local relapse were subjected to a competing risk analysis using an accelerated failure-time model with a log-logistic hazard function. Randomization to CHART ($2P = 0.005$), increasing age ($2P = 0.036$) and female sex ($2P = 0.09$) was all associated with a prolonged interval to failure. Advanced clinical stage was associated with a decreased interval to failure ($2P = 0.004$) and a significantly increased risk ($2P = 0.009$) of failing in distant rather than in local position. From this model, prognostic indices for local and distant failure were estimated for each individual patient. Competing risk analysis allows identification of patients with different failure patterns, and may provide a means of stratifying patients for intensified local or systemic therapy. © 2001 Cancer Research Campaign http://www.bjcancer.com

**Keywords**: NSCLC; CHART; competing risks; failure-specific prognostic factors

Many clinical trials employing combination of treatment modalities for medically or technically unresectable locally advanced non-small-cell lung cancer (NSCLC) have evolved in recent years (Ardizzoni et al, 1999; Ball et al, 1999). Although modest improvements in outcome have been reported, the optimal combination of these treatment modalities and the selection of patients for the treatment options from which they are most likely to benefit remain controversial (Stevens et al, 2000). A recent pattern of failure study from the Radiation Therapy Oncology Group (RTOG) for locally advanced NSCLC suggested the possibility of important differences in failure-specific outcomes by pre-treatment characteristics and the treatment assigned (Cox et al, 1999). The analysis of different modes of failures (local and distant) and their relationship to baseline variables is complicated by the presence of competing risks (Kalbfleisch and Prentice, 1980; Kramar et al, 1987; Arriagada et al, 1992). A competing risk is defined as an event, which may or may not occur during follow-up but if it occurs will prevent observing time to any other event of interest. In the case of local control, a competing risk would be to develop a distant metastasis; simply because patients have a short life expectancy after distant failure and therefore are no longer at risk for local failure.

The purpose of this study was to apply a competing risks model in order to identify factors associated with local and/or distant failure in NSCLC. This way, it should be possible to identify subgroups of patients who are more likely to benefit from either intensified local or systemic therapy. An updated database of the randomized study comparing continuous hyperfractionated accelerated radiotherapy (CHART) and conventional radiotherapy in locally advanced inoperable NSCLC was utilized. The CHART schedule was introduced at Mount Vernon Hospital, UK, in 1985 aiming to improve local tumour control by increased dose intensity using many small fractions and reduced overall treatment time. Definitive results of the trial were published first in 1997 and the mature data were analysed and published in 1999 (Saunders et al, 1999).

*Received 30 May 2001*
*Revised 5 July 2001*
*Accepted 13 July 2001*

*Correspondence to:* ÖU Ataman

### PATIENTS AND METHODS

### Study population

The database contained information on a total of 563 lung cancer patients from 13 centres entered into the CHART trial between April 1990 and April 1995. The randomization procedure was designed to produce a 60% chance of a patient receiving CHART and a 40% chance of receiving conventional radiotherapy. Patients eligible for the trial were those who had a WHO performance status of 0 or 1, and presented histologically proven, inoperable NSCLC, considered suitable for radical radiotherapy. Fourteen patients were excluded from the analysis because they did not have assessable T or N stages; thus information on all variables was available in 549 patients. The covariates considered in the modelling, the various categories and numerical scorings are shown in Table 1.

### Radiotherapy

The radiotherapy planning was identical for all patients regardless of the treatment allocated. During the first phase of the radiotherapy

**Table 1** Prognostic factors and their categories with numerical scores in the analysis group ($n$ = 549)

| Covariate | Category | Score | No. | % |
|---|---|---|---|---|
| Sex | | | | |
| | Male | 1 | 423 | 77 |
| | Female | 2 | 126 | 23 |
| Age | Continuous | | 549 | 100 |
| WHO status | | | | |
| | (0) No restriction | 1 | 224 | 41 |
| | (1) Restriction | 2 | 325 | 59 |
| T stage | | | | |
| | T1 | 1 | 47 | 9 |
| | T2 | 2 | 245 | 45 |
| | T3 | 3 | 139 | 25 |
| | T4 | 4 | 118 | 21 |
| Nodal stage | | | | |
| | N0 | 1 | 268 | 49 |
| | N1 | 2 | 74 | 13 |
| | N2 | 3 | 192 | 35 |
| | N3 | 4 | 15 | 3 |
| Clinical stage | | | | |
| | I and II | 1 | 203 | 37 |
| | IIIA and IIIB | 2 | 346 | 63 |
| Histology | | | | |
| | Squamous | 1 | 316 | 58 |
| | Other | 2 | 233 | 42 |
| Treatment | | | | |
| | CHART | 1 | 329 | 60 |
| | Conventional | 2 | 220 | 40 |
| Irradiated area | | | | |
| | 160 $cm^2$ | 1 | 187 | 34 |
| | 161–199 $cm^2$ | 2 | 185 | 34 |
| | 200 $cm^2$ | 3 | 177 | 32 |

a large volume was irradiated which included the mediastinum and the primary tumour together with a 1 cm margin. The ipsilateral hilar nodes and the paratracheal nodes but not the contralateral hilar nodes were included in the field. In the second phase, the small volume treated included the primary tumour and known nodal involvement with a 1 cm margin.

Patients randomized to conventional radiotherapy received a daily dose of 2 Gy on 5 days per week; the large volume was given 44 Gy and the small volume 16 Gy so that the total dose was 60 Gy in 30 fractions. Those randomized to CHART, received an individual dose of 1.5 Gy, given 3 times per day on each of 12 consecutive days with an interval of at least 6 hours between treatments. The large volume received 37.5 Gy in 25 fractions followed by 16.5 Gy in 11 fractions to the small volume so that the total dose was 54 Gy in 36 fractions.

### Follow-up and endpoints

Assessments were made weekly for the first 6 weeks starting from the beginning of the treatment, then at 8 and 12 weeks. Subsequently, patients attended 3 monthly until 2 years, 6 monthly to 5 years and annually thereafter. At every visit a chest X-ray was taken and the tumour status was recorded; at 6 months computerized tomography (CT) of the chest was performed. In previous publications from the CHART trial local tumour control was defined as either (a) complete disappearance of all abnormalities in a chest X-ray or CT scan, or (b) when any residual abnormality observed at 6 months remained stable for a further 6 months or more. In a competing risks analysis, this definition causes some problems. First, patients with residual abnormalities at 6 months who are not followed for 12 months or more are in effect not evaluable according to the above definition. Second, patients with residual disease after radiotherapy are considered to have failed locally at time zero. This creates a mathematical discontinuity at this point in time, which is not well modelled by a parametric survival time model as the one we use here. In the present study, time to local progression was used as an endpoint and this was defined as progression of the primary tumour within the irradiated volume detected on a chest X-ray or CT scan. Distant failure was defined as appearance of metastatic disease outside the irradiated volume.

In each patient, the time to first failure and its position (local or distant) were recorded, or, in patients without clinical progression, the time of the last follow up was used as input data (censored cases) for the analysis. All times were calculated from the date of randomization. Patients with synchronous distant and local failure were classified as failing distantly in order to be able to focus on the efficacy of the intensified local treatment.

### Competing risks

Competing risk analysis was performed using an accelerated failure-time model with a log-logistic hazard function. For each covariate, 3 hypotheses were tested:

$H_{comb}$: this is the *combined* hypothesis that covariate has no influence on either type of or time until failure.
$H_{FT}$: the covariate has no influence on *failure* type.
$H_{cond}$: the *conditional* hypothesis that the covariate does not influence time to failure given that it does not influence type of failure.

After identification of the relevant prognostic variables, a prognostic index (PI) for each individual patient for a specific failure type (FT) was calculated as:

$$PI_{FT} = \beta_{FT\ sex}.\ I(sex = female) + \beta_{FT\cdot trt}.\ I(trt = conventional) + \beta_{FT\cdot stage}.\ I(stage = III) + \beta_{FT\cdot age}.age$$

Where the $\beta$'s are the regression coefficients for the various prognostic factors for the specific failure type and 'I' is the indicator function, which can take the values 1 and 0, depending on whether a given characteristic is present or not.

The prognostic indices for each individual patient for distant and local failure were used to estimate the log-logistic (LL) failure time distribution for the patient:

$$F_{FT}(t) = 1 - S_{FT}(t) = 1 - \frac{1}{1 + (\exp(-\alpha_{FT} - PI_{FT})t)^{\psi}}.$$

Where $\alpha$ is a constant and $\psi$ is a shape parameter for the specific failure type ($\psi$ is $1/\sigma$ where $\sigma$ is the scale parameter in the output from the BMDP software package) (BMDP *User's Guide*, 1992a).

The performance of the model was first tested using standard Kaplan–Meier (KM) and log-rank methods. Further validation of the model was carried out by the calculation of the modelled failure rates at 3 years for each group and comparison of the results to the 1-KM estimates graphically by time.

 © 2001 Cancer Research Campaign

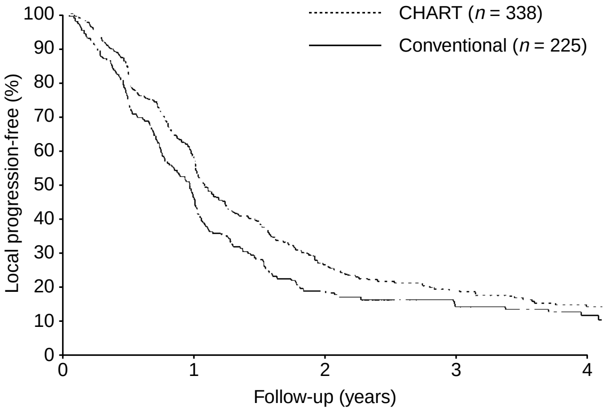

**Figure 1** Local progression-free rate in the two arms of the CHART bronchus trial. The log-rank $P$ value was 0.015

### RESULTS

Isolated local failure was the first failure in 264 patients and distant failure with or without local failure was the first failure type in 201 patients. The number of censored patients was 84. There were 252 (74%) local failures in the CHART arm and 176 (78%) local failures in the conventional arm. Local progression-free survival at 4 years was 14% for the CHART arm and 11% for the conventional arm, which is a statistically significant difference ($P$ = 0.015). The local progression-free rate is shown in Figure 1.

Nonsignificant variables ($P$ > 0.05) were excluded from the analysis in a stepwise manner and the final reduced model included only 4 variables: age, sex, clinical stage and treatment. The influence of the significant covariates on the 2 types of failures is presented in Table 2. Female gender and increasing age were associated with a prolonged time to failure. Patients receiving conventional radiotherapy failed earlier compared to the patients in the CHART arm and advanced clinical stage was associated with a relatively earlier failure.

The results of hypothesis testing are shown in Table 3 where the $H_{comb}$ was significant for both the treatment arm (2$P$ = 0.005) and the clinical stage (2$P$ = 0.004), whereas a borderline significance was observed for age (2$P$ = 0.08). The analysis of $H_{FT}$ and $H_{cond}$ reveals that treatment arm had a significant influence on time to failure but was not selective for the failure type. However advanced clinical stage was associated with a decreased interval to failure (2$P$ = 0.004) and a significantly increased risk (2$P$ = 0.009) of failing in distant rather than in local position.

The final model was used to construct prognostic indices and specific failure rates at 2 years were used to identify 4 prognostic groups. The scatter plot of the local and distant failure rates of individual patients is shown in Figure 2. The median values of the estimated failure rates at 2 years were used to define 4 prognostic groups with different risks for local and distant failure: groups with differing failure profile, Group 1 ($n$ = 173): low risk of both types of failures; Group 2 ($n$ = 103): low risk of distant metastasis, high risk of local failure; Group 3 ($n$ = 100): high risk of distant failure, low risk of local failure; Group 4 ($n$ = 173): high risk of both types of failures.

The performance of the model was first tested by standard methods and failure specific-free rates were compared using log-rank test and the results are shown in Figures 3 and 4. The local failure-free rates were 0.45 for group 1 and 0.44 for group 3 where it was only 0.32 and 0.28 for groups 4 and 2 at 2 years respectively. The log-rank $P$ value was 0.05 for local failure-free rate between 4 groups. The model was much more powerful for the distant metastasis-free rates, where the $P$ value was 0.0005 and groups 1 and 2 had distant failure-free rates of 0.58 and 0.60 at 2 years, respectively.

Modelled failure time distributions were compared graphically with 1-KM estimates over time in order to validate the model. There were close agreement between the modelled failure time distributions and 1-KM estimates in all 4 groups and Figures 5–8 show the comparison in group 1 and 4 as examples.

**Table 2** Failure-specific coefficients of prognostic variables in the final model

| Covariate | Type of failure: Distant failure Coefficient | Distant failure Standard error | Local failure Coefficient | Local failure Standard error |
|---|---|---|---|---|
| Sex | 0.1096 | 0.1799 | 0.2056 | 0.1241 |
| Age | 0.0168 | 0.0090 | 0.0076 | 0.0060 |
| Treatment | –0.0448 | 0.1545 | –0.3379 | 0.1049 |
| Clinical stage | –0.5455 | 0.1656 | –0.0316 | 0.1080 |

**Table 3** Results of hypotheses testing

| Covariate | $H_{comb}$ *P* value | $H_{FT}$ *P* value | $H_{cond}$ *P* value |
|---|---|---|---|
| Sex | 0.211 | 0.661 | 0.088 |
| Age | 0.078 | 0.398 | 0.036 |
| Treatment | 0.005 | 0.116 | 0.005 |
| Clinical stage | 0.004 | 0.009 | 0.041 |

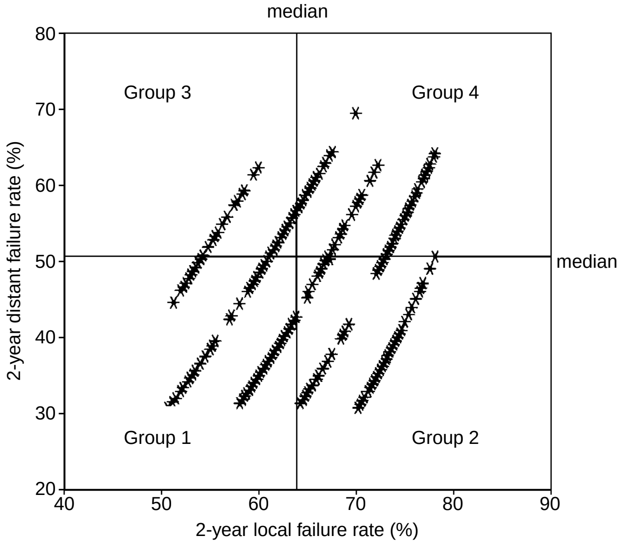

**Figure 2** Scatter plot of the estimated local and distant failure rates at 2 years for each individual patient. The median values of failure rates were used to define 4 prognostic groups with different failure pattern. Each star represents a single patient

© 2001 Cancer Research Campaign

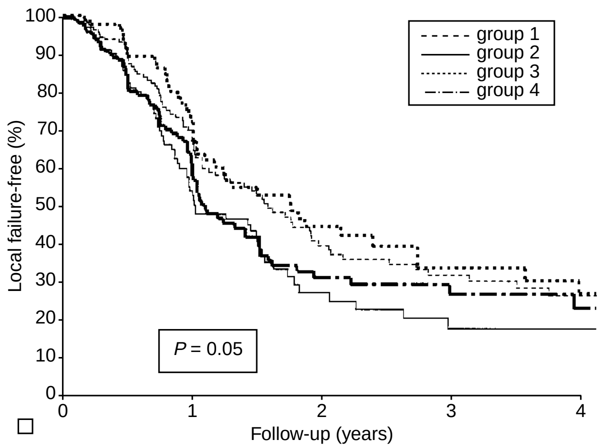

**Figure 3** Local failure-free rates compared between 4 prognostic groups using KM estimates and log-rank test. Groups 1 and 3 have higher local failure-free rates than the other 2 groups as expected from the modelling

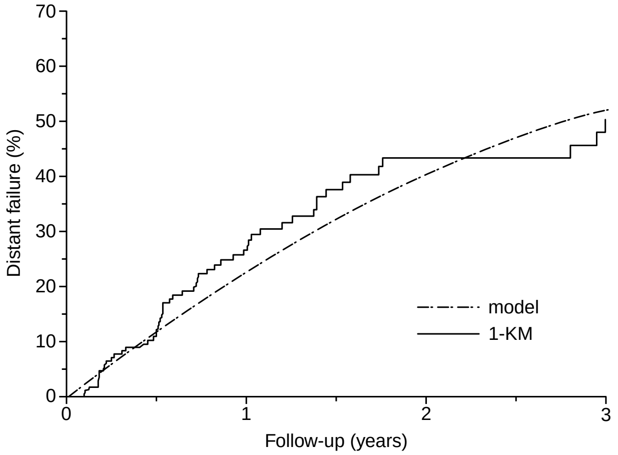

**Figure 5** Modelled distant failure rate distribution in group 1 (dashed line) compared graphically with 1-KM estimates over time

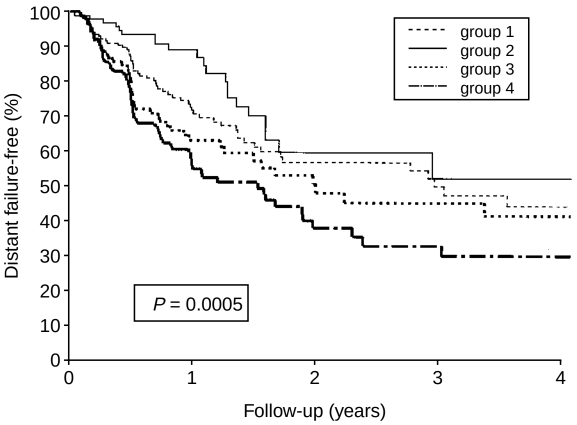

**Figure 4** Distant failure-free rates compared between the 4 prognostic groups using KM estimates and log-rank test. Groups 1 and 2 have higher distant failure-free rates than the other 2 groups as expected from the modelling

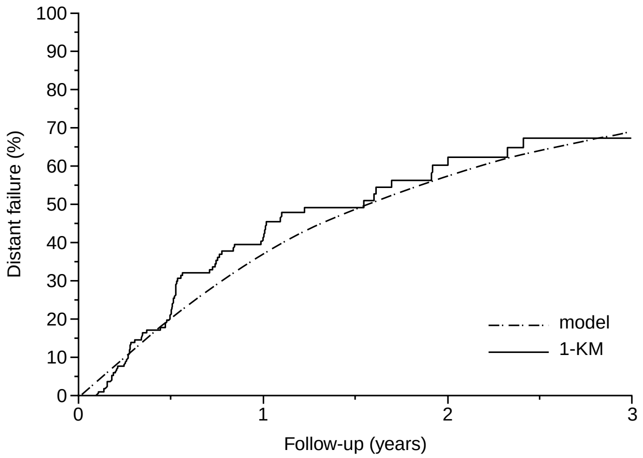

**Figure 7** Modelled distant failure rate distribution in group 4 (dashed line) compared graphically with 1-KM estimates over time

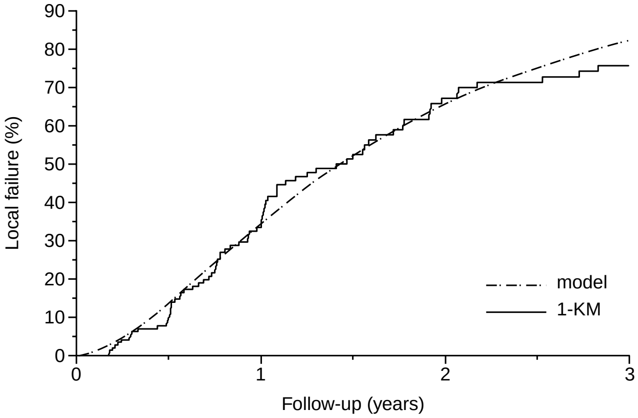

**Figure 6** Modelled local failure rate distribution in group 1 (dashed line) compared graphically with 1-KM estimates over time

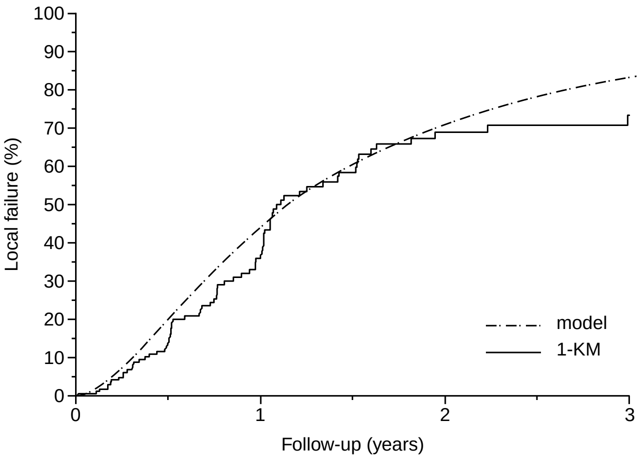

**Figure 8** Modelled local failure rate distribution in group 4 (dashed line) compared graphically with 1-KM estimates over time

*© 2001 Cancer Research Campaign*

### DISCUSSION

Competing risks analysis has been developed theoretically for more than 2 decades and is available in some of the standard statistical software packages (S-Plus, 1991; SAS, 1993). Nevertheless, this type of analysis has found relatively limited practical use in prognostic studies in cancer research. The conceptual advantage of competing risks analysis is that it takes both the type of failure and the time of failure into account. In patients with inoperable NSCLC both local and distant failures are very common. These are obviously competing events because the occurrence of either one of them is likely to lead to the death of the patient and thereby to termination of the time at risk for developing the other failure type. The aim of the present study was to evaluate how disease, patient and treatment characteristics influence the time to local and distant failure in NSCLC. Although many studies of prognostic factors have been conducted in NSCLC, they have not analysed failure-specific outcomes in the competing risks setting (Albain et al, 1991; Takigawa et al, 1996).

We applied the competing risk analysis first proposed by Lagakos (Lagakos, 1978) as implemented in the BMDP statistical software package (BMDP *User's Guide*, 1992b). This is an accelerated failure time model where the effect of a statistically significant covariate will be to prolong or shorten the time until failure depending on the sign of the regression coefficient (see Table 2). These signs must be considered in relation to the scoring of covariates shown in Table 1. A positive regression coefficient means that increasing values of the covariate will *increase* time to failure and a negative regression coefficient means that increasing values of the covariate will *decrease* the time to failure. (This convention for the signs is in contrast to the convention used with the Cox Proportional Hazards Model, where a positive regression coefficient indicates a negative effect on failure times with increasing values of the corresponding covariate.) A recent study, using the accelerated failure time model with a log-normal failure time distribution, is the analysis of failure patterns in primary breast cancer by Chapman and colleagues (Chapman et al, 1999). Here, we used a log-logistic failure time distribution, which is similar to the log-normal distribution but has the computational advantage that the cumulative distribution function has a simple analytic form (Bentzen et al, 1989).

In the present analysis, the time to failure increased with increasing age (continuous variable) as shown by the positive regression coefficient (Table 2). Another example is treatment which was coded CHART = 1 and CONV = 2. Here, the patients receiving conventional radiotherapy failed earlier than those receiving CHART, for both types of failures. This effect was more pronounced for local rather than distant failure as seen from the magnitude of the 2 regression coefficients (Table 2).

The competing risks analysis applied here also provides a framework for testing 3 hypotheses concerning the importance of each covariate for specific failure types. The results of these tests are shown in Table 3. $H_{FT}$ tests whether the regression coefficients for local and distant failure are significantly different. Take the effect of patient's age as an example. Looking at the regression coefficients in Table 2, age is seen to prolong the time to both distant and local failure. The magnitude of these regression coefficients should be seen in relation to their standard errors. Clearly, these 2 regression coefficients are not statistically significantly different and this is reflected by the $P$ value for rejecting $H_{FT}$, which is 0.4 (Table 3). In this case, the conditional hypothesis, $H_{cond}$, that age has no influence on time to failure given that it has no influence on type of failure, is relevant to test, and this can be rejected with $P = 0.036$ in the present analysis. Thus, increasing age prolongs the time to failure but has a similar effect for the 2 competing failure types. $H_{FT}$ could only be rejected for clinical stage ($2P = 0.009$), which means that this factor had a significantly different effect on distant and local failure times. In this case, the conditional hypothesis, $H_{cond}$, is clearly not relevant. The combined hypothesis, $H_{comb}$, could be rejected ($2P = 0.004$), i.e. clinical stage is associated with a decreased time to failure and discriminates between the 2 failure types. None of the other covariates were significantly associated with a specific type of failure, although from the regression coefficients it is seen, that patients receiving CHART had a numerically larger prolongation of the time to local failure than the time to distant failure.

The final model was used to construct prognostic indices and specific failure rates at 2 years and these were used to identify 4 groups with different failure pattern. The scatter plot of the local and distant failure rates of individual patients are shown in Figure 2 where each patient is represented by a star. The 'streaky' appearance of the patients' predicted failure rates results from the continuous age parameter.

Failure rates in the 4 prognostic groups were evaluated by KM estimates using the log-rank test for comparison of groups and the 4 groups were shown to vary in the risk of the 2 failure types. Group 3 and 4 had a higher risk of failing distantly and the distant failure-free rates were significantly lower ($2P = 0.0005$) than in the other 2 groups. Likewise, group 2 and 4 had a higher risk of failing locally and again local failure-free rates were significantly lower ($2P = 0.05$) than in the other two groups (Figures 3 and 4).

An informal test of the model fit to the data was performed graphically by plotting the 1-KM estimates and the failure rates estimated from the log-logistic model as a function of time. These plots showed a close agreement between the model and the empirical estimates as shown by the examples given in Figures 5–8.

Classification of patients according to a prognostic index combining multiple prognostic factors has been used extensively with the Cox model (e.g. Bentzen et al, 1988). In patients with NSCLC, Wigren recently applied this type of index to discriminate between patients with a poor and a relatively good survival (Wigren, 1997). We have generalized this idea in a failure-type-specific competing risks setting. This is potentially a powerful method for further stratification of patients into groups requiring different therapeutic strategies.

Three recent papers from the RTOG group (Scott et al, 1997; Komaki et al, 1998; Werner-Wasik et al, 2000) used recursive partitioning analysis (RPA) for defining prognostic subgroups of patients with locally advanced inoperable NSCLC. The RPA method creates branches of the prognostic factors from the stem of all patients (Ciampi et al, 1988). The entire patient population is partitioned into subclasses according to the variable producing the most significant survival difference to form final prognostic classes. One of these studies was concerned with patterns of failure and aimed to identify groups of patients with different failure rates (Komaki et al, 1998). Four RPA classes were constructed from Kaplan–Meier survival estimates. For each of these, the frequency distribution of the site of first failure was presented. Clearly, this is not a competing risks approach, and in fact the 4 RPA classes showed an almost counterintuitive trend towards more disease failures in the best prognostic class. In the worst prognostic group, 58% of the patients died without disease progression as compared

*© 2001 Cancer Research Campaign*

with 27% in the best group. It could seem that patients with a long life expectancy survived long enough to have a detectable disease progression. However, it is difficult to interpret the findings of this analysis in terms of failure patterns.

In conclusion, we used a competing risks model to assess the effects of clinico-pathological factors on different types of first recurrence after radical radiotherapy for locally advanced NSCLC. We have shown that for stage III disease, distant failure rates are relatively higher than the local failure rates. Thus, intensified local treatment may not suffice to improve therapeutic outcome in this group of patients. While the present analysis identified 4 subgroups with significantly different failure pattern, there is still a high failure rate at both local and distant position in patients with inoperable NSCLC.

The literature is rapidly expanding in the field of biological markers and their potential role as prognostic or predictive factors remains to be tested (Sørensen and Østerlind, 1999). It is possible that further biological characterization of these tumours in a competing risk analysis would enable us to predict the risk of specific types of failure with improved precision. This in turn could form a rational basis for individualization of treatment prescription.

### ACKNOWLEDGEMENTS

This research was funded by the Scott of Yews Trust and the Gray Laboratory Cancer Research Trust and by the Radiation Oncology Department of the Dokuz Eylul University Medical School. We wish to acknowledge the great efforts of all members of the CHART Steering Committee: A Barrett (Chairman), D Coyle, B Cottier, AM Crellin, P Dawes, S Dische, MF Drummond, C Gaffney, D Gibson, A Harvey, JM Henk, T Herrmann, B Littbrand, J Littler, F Macbeth, DAL Morgan, H Newman, MKB Parmar, AG Robertson, M Robinson, RI Rothwell, MI Saunders, RP Symonds, JS Tobias, MJ Whipp, H Yosef.

### REFERENCES

Albain KS, Crowley JJ, LeBlanc M and Livingstone RB (1991) Survival determinants in extensive-stage non-small cell lung cancer: the Southwest Oncology Group experience. *J Clin Oncol* **9**(9): 1618–1626

Ardizzoni A, Grossi F, Scolaro T, Giudici S, Foppiano F, Boni L, Tixi L, Cosso M, Mereu C, Battista Ratto G, Vitale V and Rosso R (1999) Induction chemotherapy followed by concurrent standard radiotherapy and daily low-dose cisplatin in locally advanced non-small-cell lung cancer. *Br J Cancer* **81**(2): 310–315

Arriagada R, Kramar A, Le Chevalier T, De Cremoux H (1992) Competing events determining relapse-free survival in limited small-cell lung carcinoma. *J Clin Oncol* **10**: 447–451

Ball D, Bishop J, Smith J, O'Brien P, Davis S, Ryan G, Olver I, Toner G, Walker Q and Joseph D (1999) A randomised phase III study of accelerated or standard fraction radiotherapy with or without concurrent carboplatin in inoperable non-small cell lung cancer: final report of an Australian multi-centre trial. *Radiother Oncol* **52**: 129–136

Bentzen SM, Poulsen HS, Kaae S, Myhre Jensen O, Johansen H, Mouridsen HT, Daugaard S and Arnoldi C (1988) Prognostic factors in osteosarcomas A regression analysis. *Cancer* **62**: 194–202

Bentzen SM, Thames HD, Travis EL, Kian Ang K, Van Der Scheren E, Dewit L and Dixon DO (1989) Direct estimation of latent time for radiation injury in late responding normal tissues: gut, lung, and spinal cord. *Int J Radiat Oncol Biol Phys* **55**(1): 27–43

*BMDP User's Guide* (1992a), Version 7, ed. Dixon WJ. pp 825–864, Wiley

*BMDP User's Guide* (1992b), Version 7, ed. Dixon WJ. Appendix B5, pp 1392–1396 Wiley

Chapman JW, Fish EB and Link MA (1999) Competing risk analyses for recurrence from primary breast cancer. *Br J Cancer* **79**(9–10): 1508–1513

Ciampi A, Lawless JF, Mc Kinney SM and Singhal K (1988) Regression and recursive partition strategies in the analysis of medical survival data. *J Clin Epidemiol* **41**(8): 737–748

Cox JD, Scott CB, Byhardt RW, Emami B, Russell AH, Fu KK, Parliament MB, Komaki R and Gaspar LEW (1999) Addition of chemotherapy to radiation therapy alters failure patterns by cell type within non-small cell carcinoma of lung (NSCLC): Analysis of Radiation Therapy Oncology Group (RTOG) trials. *Int J Radiat Oncol Biol Phys*, **43**(3): 505–509

Kalbfleisch JD and Prentice RL (1980) *The statistical Analysis of Failure Time Data*, pp 21–38 and pp 179–188. John Wiley: New York

Komaki R, Scott CB, Byhardt R, Emami B, Asbell SO, Russell AH, Roach M, Parliament MB and Gaspar LE (1998) Failure patterns by prognostic group determined by recursive partitioning analysis (RPA) of 1547 patients on four radiation therapy oncology group (RTOG) studies in inoperable nonsmall-cell lung cancer (NSCLC). *Int. J. Radiat Oncol Biol. Phys* **42**(2): 263–267

Kramar A, Pejovic MH and Chassagne D (1987) A method of analysis taking into account competing events: Application to the study of digestive complications following irradiation for cervical cancer. *Statist Med* **6**: 785–794

Lagakos SW (1978) A covariate model for partially censored data subject to competing causes of failure. *Appl Statist* **27**(3): 235–241

*SAS/STAT User's Guide* (1993) Version 6, 4th ed, SAS Institute: Cary NC, pp 997–1025

Saunders M, Dische S, Barrett A, Harvey A, Griffiths G and Parmar M (on behalf of CHART Steering committee) (1999) Continuous, hyperfractionated, accelerated radiotherapy (CHART) versus conventional radiotherapy in non-small cell lung cancer: mature data from the randomised multicentre trial. *Radiother Oncol* **52**: 137–148

Scott C, Sause WT, Byhardt R, Marcial V, Pajak TF, Herskovic A and Cox JD (1997) Recursive partitioning analysis of 1592 patients on four Radiation Therapy Oncology Group studies in inoperable non-small cell lung cancer, *Lung Cancer* **17**(suppl 1): 59–74

*S-Plus 4 Guide to Statistics* (1991) Data analysis products division, Math Soft: Seattle Wa, pp 697–710

Sørensen JB and Østerlind K (1999) Prognostic factors: from clinical parameters to new biological markers. In *Progress and perspective in the treatment of lung cancer* Van Houtte P, Klastersky J, Rocmans P (Eds). pp 1–21, Springer

Stevens CW, Lee JS, Cox J and Komaki R (2000) Novel approaches to locally advanced unresectable non-small cell lung cancer. *Radiother Oncol* **55**: 11–18

Takigawa N, Segawa Y, Okahara M, Maeda Y, Takata I, Kataoka M and Fujii M (1996) Prognostic factors for patients with advanced non-small cell lung cancer: univariate and multivariate analyses including recursive partitioning and amalgamation. *Lung Cancer* **15**: 67–77

Werner-Wasik M, Scott C, Cox JD, Sause WT, Byhardt RW, Asbell S, Russell A, Komaki R and Lee JS (2000) Recursive partitioning analysis of 1999 Radiation Therapy Oncology Group (RTOG) patients with locally advanced non-small cell lung cancer (LA-NSCLC); identification of five groups with different survival. *Int J Radiat Oncol Biol Phys* **48**: 1475–1482

Wigren T (1997) Confirmation of a prognostic index for patients with inoperable non-small cell lung cancer. *Radiother Oncol* **44**: 9–15

*© 2001 Cancer Research Campaign*
